# Isokinetic Strength Profile of Elite Female Handball Players

**DOI:** 10.1515/hukin-2015-0128

**Published:** 2015-12-30

**Authors:** Zuzana Xaverova, Johannes Dirnberger, Michal Lehnert, Jan Belka, Herbert Wagner, Karolina Orechovska

**Affiliations:** 1 Faculty of Physical Culture, Palacky University in Olomouc, Czech Republic; 2 Department of Sport Science and Kinesiology, University of Salzburg, Hallein-Salzburg, Austria

**Keywords:** H/Q ratios, bilateral deficit, torque, strengthening

## Abstract

Systematic assessment of muscle strength of the lower extremities throughout the annual training cycle in athletes is crucial from a performance perspective for the optimization of the training process, as well as a health perspective with regard to injury prevention. The main aim of the present study was to determine isokinetic muscle strength of the knee flexors and extensors in female handball players at the beginning of a preparatory period and to assess whether there were any differences between players of different performance levels. The performance level was expressed by means of membership of the Women’s Junior National Handball Team (JNT, n=8) or the Women’s National Handball Team (NT, n=9). The isokinetic peak torque during concentric and eccentric single-joint knee flexion and extension was measured at angular velocities of 60, 180, 240°/s (concentric) and 60°/s (eccentric). The Mann-Whitney test showed no significant differences in the peak torques or ipsilateral ratios between the two groups. The bilateral force deficit (BFD) for concentric extension at 240°/s was significantly higher in the JNT compared with the NT (p=0.04; d=1.02). However, the results of individual evaluation show that the BFD was more frequent in the NT in most measurements. A high BFD was evident in the eccentric mode in both groups highlighting a need for particular strengthening. With regard to low strength ratios a prevention programme should be suggested for both observed groups of professional female handball players to reduce the risk of injury.

## Introduction

Handball is a high-intensity Olympic sport with an intermittent game character and frequent body contacts requiring high demands for a degree of variability of locomotor actions such as repeated accelerations, sprints, and rapid changes in direction, stops and jumps (Bencke et al., 2013). In these actions, the knee extensors and knee flexors are important prime movers. The knee extensors are involved in running and jumping – during the take-off phase, as well as during landing in the deceleration phase, where they control knee flexion during the eccentric action. The knee flexors affect stride length and stabilize the knee joint during acceleration, changes in moving direction, deceleration and landing (Stølen et al., 2005).

In handball, ACL injuries are common and frequent, with, for example, an incidence of more than 4.5% in Norwegian elite female handball players (Myklebust et al., 1997). Lohmander et al. (2007) confirmed that the highest incidence of ACL injuries occurred in adolescents playing sports that involved pivoting, such as football, basketball and handball. ACL injuries in handball mostly occur during non-contact high-speed side-cutting movements and more frequently during competition than training (Myklebust et al., 1998). Moreover, ipsilateral (e.g. knee flexors vs. extensors) or bilateral (e.g. the right vs. left flexors) imbalances lead to an increased probability of hamstring and other soft tissue knee joint injuries (Dauty et al., 2003; Stastny et al., 2014). It was also reported that top- level female handball players faced twice the risk of cruciate ligament injury than males and the risk was even increased by the level of competition or years of training (Myklebust et al., 1997, 1998).

For the reasons mentioned above, systematic assessment of the isokinetic muscle strength of the lower extremities throughout the annual training cycle is crucial from a performance perspective for the optimization of the training process, as well as a health perspective with regard to injury prevention. Assessment by isokinetic dynamometry has been considered as the gold standard to provide an objective approach to diagnostics and simpler quantification of muscle strength. Most studies in handball female players have concentrated on concentric measurements. Based on these measurements, ipsilateral imbalances were described by the conventional hamstring to quadriceps ratio (H/QCONV) (Andrade et al., 2012; Lund-Hanssen et al., 1996). However, the conventional ratio has experienced much criticism in recent years as not being functional (Coombs and Garbutt, 2002; Dauty et al., 2003). Actually, co-activation of the knee flexors and extensors is known to occur and take place through opposing contraction modes, so the functional role of the hamstrings in many sports and physical activities (e.g. running) is to decelerate the lower leg during rapid and forceful concentric contractions of the knee extensors with a protective eccentric contraction (Coombs and Garbutt, 2002). Increased antagonistic activation of the hamstrings reduces redundant tension of the anterior cruciate ligament (ACL), and should help avoid overextension by decelerating the leg prior to full extension and stabilizing the knee joint throughout the range of motion (Aagaard et al., 1998). Therefore, Coombs and Garbutt (2002) strongly recommend to evaluate eccentric strength too and to additionally express imbalances using a functional ratio Hecc/Qcon (H/QFUNC). However, the only study in handball players including eccentric measurements was performed in males (Carvalho et al., 2014). There is no study in females, although various studies have shown that the development of eccentric strength is different in males and females, possibly because the actions have separate neural control mechanisms (Enoka, 1996). Therefore, the main aim of the present study was to determine isokinetic muscle strength of the knee flexors and extensors in female handball players at the beginning of a preparatory period and to assess whether there were any differences between players of a different performance level. Due to a research deficit, not only concentric absolute values and concentric ratios, but also eccentric and functional values were considered.

## Material and Methods

### Participants

The study group consisted of 17 elite female handball players (Table 1) who competed in the International Czech-Slovak Inter League. This group was further divided into two groups: the JNT (n=8) consisted of players who participated in the Women’s Junior National Handball Team during the past three years and the NT (n=9) consisted of players who participated in the Women’s National Handball Team during the past three years. All participants had previously experienced isokinetic testing. The exclusion criteria were: a) self-reported health problems concerning pain during the testing procedure (n=2); b) previous knee injury (n=2); c) playing position of a goalkeeper (n=2). The players self-reported their preferred leg for kicking a ball as their dominant leg (DL), and simultaneously self-reported their contralateral leg as the take-off leg for a jump shot as non-dominant (NL). There were 14 players with the right dominant leg and 3 with the left dominant leg. All participants were asked to maintain their normal diet and refrain from training for 48 hours prior to each testing session. Written informed consent was provided by the participants, and the testing protocol was approved by the Ethical Committee of the Faculty of Physical Culture at the Palacky University in Olomouc in accordance with the ethical standards of the Declaration of Helsinki (1983).

### Procedures

**Figure 1:**
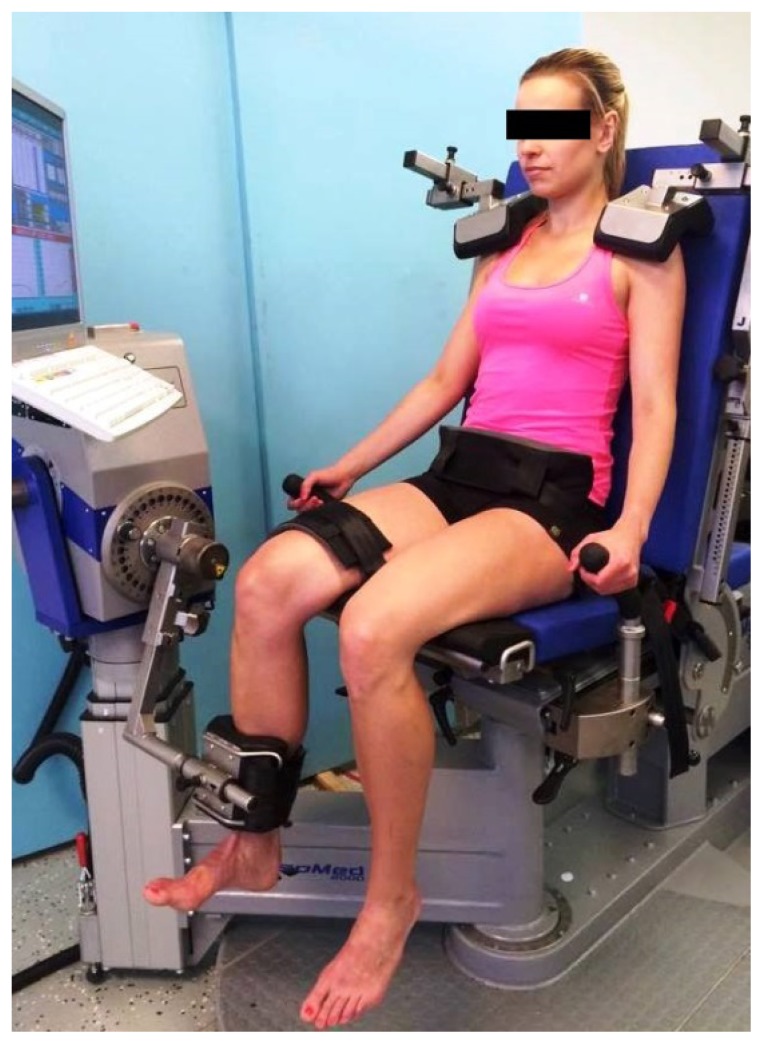
Testing position on the IsoMed 2000 dynamometer

Bilateral isokinetic strength of the knee flexors and extensors was measured using the IsoMed 2000 isokinetic dynamometer (D. & R. Ferstl GmbH, Hemau, Germany). The reliability of measurement was confirmed by Dirnberger et al. (2012). Before testing the subjects completed a non-specific warm-up on a stationary bicycle ergometer for 6 min at a self-regulated low to moderate intensity, followed by 10 min of dynamic stretching that targeted the main muscle groups involved during testing, and 8 squats with progressive descending. The warm-up routine was performed under the supervision of a researcher. The participants were then comfortably seated on an adjustable dynamometer chair with the hip joint at about 75° (0°=full extension). The pelvis and the thigh of the right leg, which was tested first, were fixed by means of straps, and the shoulders were fixed in the ventral-dorsal and cranial-caudal direction by shoulder pads. For further stabilisation of the upper body, the subjects were instructed to hold the handgrips located at the side of the seat during all testing efforts. The axis of rotation of the dynamometer was aligned with the axis of rotation of the right knee using the lateral femoral epicondyle as reference. The lever arm of the dynamometer was fixed to the distal part of the shin so that the lower edge of the shin pad was located ≈ 2 cm over the medial apex malleolus. After fixation, a static gravitational correction was applied. The range of motion (ROM) for testing was set from 10 to 90° of knee flexion (0°=full knee extension). Figure 1 shows the final testing position as described above.

Testing of the knee extensors and flexors was conducted at an angular velocity of 60°/s in concentric/concentric and eccentric/eccentric reciprocal actions and angular velocities of 180 and 240°/s in concentric/concentric reciprocal actions. The slow angular velocity was performed before the high velocities to reduce the risk of injury (Ayala et al., 2012). Concentric action preceded eccentric action. Between the single tests there was a rest period of 2 min. Prior to each test, the subjects performed 4–5 submaximal practice trials as a specific warm-up to become acquainted with the requirements of the test. After the warm-up, the subjects were instructed to extend/flex the knee with maximum intensity throughout the entire range of motion in each of the subsequent testing repetitions. Actual testing at each velocity consisted of a set of 4 reciprocal repetitions. The players were notified by a verbal countdown and accompanied by strong verbal encouragement and visual online feedback in order to ensure maximum effort. After testing the right leg, the left leg was tested according to the same procedure, during which individual settings were automatically activated, rechecked and adjusted if necessary. All testing was performed by the same experienced examiner. In agreement with the recommendations by Aagaard et al. (1998) and Dervišević and Hadzic (2012) the monitored parameter was PT, which was also normalized for body weight and expressed as PT/kg; moreover, absolute PT was further used to determine the conventional H/Q ratio (H/Q_CONV_) and functional H/Q ratio (H/Q_FUNC_). Bilateral imbalances are represented by the bilateral force deficit (BFD) of the maximum force exerted between the legs with regard to the stronger leg according to Maly et al. (2015) as follows:

$$BFD\;(\%)=\frac{\text{Higher PT}-\text{Lower PT}}{\text{Higher PT}}*100$$

### Statistical Analysis

Descriptive statistics included mean ± standard deviation for all variables. The Mann-Whitney test for independent samples was used for evaluation of the differences in the mean values between the NT and the JNT. Moreover, effect sizes were assessed by Cohen’s *d (d* ≥ 0.20 and < 0.50 small effect, ≥ 0.50 and < 0.80 medium effect, and ≥ 0.80 large effect), (Cohen, 1988). All statistics were performed using SPSS V.20 (SPSS Inc., Chicago, Illinois, USA) and Microsoft Excel 2010 (Microsoft Corp., Redmond, Washington, USA). Statistical significance was set at *p*<0.05. Additionally, specifically for each group, the frequency of the following risk border values was evaluated: H/Q_CONC_ < 0.6, H/Q_FUNC_ < 0.7 at a velocity of 60°/s and for BFD as ≥ 10 (equal or higher than 10 but lower than 15)/≥ 15 (equal or higher than 15 but lower than 20)/≥ 20 (equal or higher than 20) % in all testing velocities and modes.

## Results

Absolute and relative peak torque values of the knee flexors and extensors in the DL and the NL according to the performance level are reported in Table 2. The ipsilateral ratio represented by the H/Q_CONV_ and the H/Q_FUNC_ on the DL and the NL, the BFD and the frequency of risk border values in individual evaluation according to the performance level are reported in Table 3.

## Discussion

The study enabled a comparison of isokinetic concentric and eccentric peak torques of the knee flexors and extensors and their bilateral and ipsilateral imbalances in different performance levels in female handball players.

### Peak torque

Muscular strength is one of the most important physical components of game performance in handball. The reported normalized average concentric PT at a velocity of 60°/s regardless of the group division and limb dominance in our study is similar to the values of female handball players of elite to second division reported by Andrade et al. (2012), but lower than the values reported by Lund-Hanssen et al. (1996). In our study no significant differences were found in isokinetic muscle strength of the knee flexors and extensors between the groups considering PT, both absolute and relative. This finding can be surprising, however, it is in accordance with the findings of studies in other team sports. Stocker et al. (1996) reported similarities in normative PT values between teams of players of various ages and skill levels in Intercollegiate Athletics female volleyball athletes. Similarly, no age-dependent increases in isometric or isokinetic muscle strength from 15 to 30 years of age in a healthy population in both males and females were reported (Harbo et al., 2012). However, Hadzic et al. (2010) reported significant differences in normalized concentric and eccentric PT in the right flexors at a velocity of 60°/s between various performance levels in male volleyball players with higher values in international players in comparison with 1^st^ and 2^nd^ division players.

### Bilateral imbalances

Bilateral muscle strength comparison is based on the premise that strength of compared muscle groups should be balanced. If this balance is disturbed, the muscle strength deficit incidence can be associated with increased susceptibility to weaker limb injury (Dauty et al., 2003). The relationship between bilateral muscle strength imbalances and an injury incidence is not clear. Nevertheless, it has been generally accepted that bilateral imbalances lower than 10% are within norms and muscle strength can be fully produced (Dauty et al., 2003). A difference higher than 15–20% is considered as a predisposition for injury and can inform about persisting weakness as a consequence of previous injury (Wrigley, 2000). According to Dauty et al. (2003), bilateral asymmetry between hamstring muscles higher than 10% should result in strengthening in order to decrease this difference.

While only a single result shows a significantly higher BFD in the JNT in concentric extension at a velocity of 240°/s in comparison with the NT, individual evaluation shows an opposite trend. During individual evaluation, we recorded the BFD ≥ 10% in 12 players, i.e. 71% (6 from the NT and 6 from the JNT), ≥ 15% in 11 players, i.e. 65% (7 from the NT and 4 from the JNT) and ≥ 20% in 6 players, i.e. 50% (5 from the NT and 1 from the JNT) at least in one velocity or mode. The highest values were recorded in the eccentric mode of action in both groups.

According to the results of individual evaluation we can assume that the BFD is more frequent in the NT in most measurements, which could be caused by long-term unilateral orientation of handball specific skills. Also, a higher BFD was evident in the eccentric mode of action in both groups highlighting a need for particular strengthening to compensate for unilateral overload because eccentric strength is an important indicator of safe functional and sport-specific tasks execution (Dauty et al., 2003).

The reported BFD might be a risk factor of injury as handball-specific movements like landing, jump shooting, drop jumping or side-cutting are usually abrupt and explosive with a very large angular change of directions (Bencke et al., 2013) and therefore, claim demands for eccentric strength. Although no relationship between leg dominance and the probability of sustaining a non-contact ACL injury was reported, higher prevalence in female athletes was found in left ACL tears (Negrete et al., 2007). However, the BFD identified by isokinetic dynamometry may not correlate with the BFD of muscular strength production during the take-off in various types of vertical jumps (Maly et al., 2015).

### Ipsilateral imbalances

We found no significant differences in ipsilateral ratios represented by the H/Q_CONV_ and H/Q_FUNC_ ratio between the NT and the JNT. This finding is not in line with the fact that a higher risk of injury is associated with higher experience levels (Myklebust et al., 1997) when ipsilateral ratios, both conventional and functional, are taken into account. The H/Q_CONV_ ratio in our study at a velocity of 60°/s, considered the most valid for strength deficit indication (Houweling et al., 2009), is lower in comparison with the generally accepted border value of 0.6 for increased probability of hamstring and ACL injury. During individual evaluation, we recorded H/Q_CONV_ < 0.6 in 10 players (5 from the NT and 5 from the JNT) on the DL and the NDL.

On the basis of a retrospective analysis of the training process we hypothesise that in the monitored handball club in the senior category, the approach applied to strength training of the lower limbs does not provide sufficient stimuli for further muscle strength development, especially when hamstring muscles are taken into account. The reason is that at this level only well-periodized resistance training can ensure a further increase or maintain muscle strength and power at a stable level (Lorenz et al., 2010). With regard to the finding that the reported average H/Q_CONV_ value of 0.57 in our handball players regardless of group division and limb dominance is similar to the value of 0.56 reported by Andrade et al. (2012) and Lund-Hanssen et al. (1996) in female handball players, we believe that our assumption mentioned above applies to more female handball clubs. As a consequence, we can also assume that the muscle loading patterns experienced during traditional handball training in females produce quadriceps-dominant athletes as Iga et al. (2009) reported in a case of traditional youth male soccer training.

Comparing strength ratios across velocities, these ratios seem to increase with increasing velocities. This is most likely due to the fact that hamstring muscles are characterised by relatively faster muscle fibres in comparison with quadriceps muscles (Garrett et al., 1984). The pattern of increased H/Q_CONV_ ratios with increasing velocities found in our study is in line with former studies of handball female players (Andrade et al., 2012; Lund-Hanssen et al., 1996) and also with studies of elite male soccer players (Maly et al., 2014). However, this pattern is contrary to the findings by Aagaard et al. (1998), who reported that the H/Q_CONV_ ratio was insensitive to changes at velocities between 30º/s and 240º/s in track and field athletes and therefore, stated that the contractile force-length properties and force-velocity properties of the agonist-antagonist muscle synergists of the knee joint were not reflected by the H/Q_CONV_ ratio.

The H/Q_CONV_ ratio has experienced much criticism in recent years. With regard to this, we also evaluated functional knee conditions at a velocity of 60°/s represented by the H/Q_FUNC_ ratio. The H/Q_FUNC_ ratio at this velocity indicates the maximum possibility of the hamstrings breaking function, and is used for the co-activation of the flexors and extensors estimation (Aagaard et al., 1998). The value of the H/Q_FUNC_ ratio is influenced by the joint angle, individual differences in muscle fibres distribution, sport type specifics (Aagaard et al., 1998; Coombs and Garbutt, 2002) and a hip abductor strength ratio (Stastny et al., 2015a; Stastny et al., 2015b). It has been suggested that the H/Q_FUNC_ ratio in a range of 0.7–1.0 is an indicator of adequate joint stability (Aagaard et al., 1998; Ayala et al., 2012; Dauty et al., 2003). Regardless of group division and limb dominance, the reported average H/Q_FUNC_ ratio value is 0.65 in our study and is below this limit in both legs. During individual evaluation, we recorded H/Q_FUNC_ < 0.7 in 12 players, i.e. 71% (6 from the NT and 6 from the JNT) on the DL and/or the NDL at velocity of 60°/s. The incidence of an insufficient value of the ratio was 10 for the DL and 11 for the NDL.

Since a low ipsilateral ratio represented by the H/Q_CONV_ and H/Q_FUNC_ has been considered an intrinsic factor of injury, our findings suggest, in accordance with Andrade et al. (2012) and Aagaard et al. (1998), that female handball players would benefit from a hamstring strengthening programme, especially with eccentric contractions. Designing a training programme should incorporate medial hamstring strengthening to match the external outward rotating knee moments and knee valgus moments (Bencke et al., 2013).

Although the research has reached its aim, there is a limitation of the study in the small amount of included players. This makes the conclusions impossible for generalization and the fact has to be taken into consideration in data interpretation.

## Conclusions

Regardless of group division, the results of the present study concerning ipsilateral as well as bilateral ratios indicate hamstring weakness and predisposition to acute knee injury in female handball players. To overcome this risk implementing a prevention programme is recommended.

In prospective studies, we suggest an assessment of seasonal variations in PT, ipsilateral ratios and bilateral imbalances during various training phases in an annual training cycle. Unless seasonal assessment is possible to conduct, we recommend to perform testing at least at the beginning of the preparatory period for identification of possible muscle strength imbalances that could be reduced by appropriate strength training programmes. Eccentric assessment should be also incorporated in the evaluation for a better understanding of strength development and adaptations.

## Figures and Tables

**Table 1:** Description of groups according to the performance level

Variable	Women’s National Team (n=9)	Junior National Team (n=8)
Age (years)	26.2 ± 3.3	20.1 ± 1.4
Body Mass (kg)	71.7 ± 10.8	69.7 ± 11.9
Body Height (m)	1.70 ± 0.05	168.8 ± 6.8
Training experience (years)	17.3 ± 3.7	11.5 ± 2.7

**Table 2:** Absolute and relative Peak torque (Nm, Nm/kg) of the knee flexors and extensors in the dominant and non-dominant leg according to the performance level (means ± SD)

				Women’s National Team		Junior National Team	
				PT	PT/kg	PT	PT/kg
				
Extensors	conc	60	DL	154.9 ± 20.2	2.2 ± 0.3	160.7 ± 16.3	2.3 ± 0.3
			NL	156.6 ± 31.2	2.2 ± 0.3	161.5 ± 13.8	2.4 ± 0.3
		180	DL	116.2 ± 13.0	1.6 ± 0.2	120.6 ± 13.9	1.8 ± 0.2
			NL	118.1 ± 14.8	1.7 ± 0.2	114.3 ± 11.5	1.7 ± 0.2
		240	DL	109.9 ± 12.7	1.6 ± 0.2	112.3 ± 15.5	1.6 ± 0.2
			NL	109.6 ± 13.4	1.6 ± 0.2	107.3 ± 11.6	1.6 ± 0.3
	ecc	60	DL	101.0 ± 15.2	1.4 ± 0.3	106.5 ± 25.8	1.5 ± 0.3
			NL	103.4 ± 17.1	1.4 ± 0.2	99.7 ± 26.7	1.4 ± 0.4
Flexors	conc	60	DL	87.7 ± 12.4	1.2 ± 0.2	93.5 ± 14.8	1.4 ± 0.2
			NL	88.2 ± 15.7	1.2 ± 0.2	90.7 ± 11.1	1.3 ± 0.1
		180	DL	80.1 ± 9.0	1.1 ± 0.2	78.1 ± 17.8	1.1 ± 0.2
			NL	75.7 ± 11.8	1.1 ± 0.2	76.1 ± 16.2	1.1 ± 0.2
		240	DL	71.6 ± 8.9	1.0 ± 0.2	78.2 ± 18.5	1.1 ± 0.2
			NL	70.3 ± 12.6	1.0 ± 0.2	74.9 ± 15.9	1.1 ± 0.2
	ecc	60	DL	195.3 ± 45.4	2.8 ± 0.8	208.6 ± 44.7	3.0 ± 0.6
			NL	208.4 ± 48.2	2.9 ± 0.6	196.1 ± 33.5	2.9 ± 0.5

PT – peak torque, PT/kg – normalized PT for body weight, conc – concentric action; ecc – eccentric action; 60, 180, 240°/s – angular velocity, DL – dominant leg, NL – non-dominant leg

**Table 3:** Ipsilateral ratio (H/Q_CONC_ and H/Q_FUNC_) and bilateral force deficit (BFD) according to the performance level (means ± SD)

			Women’s National Team	*frq*			Junior National Team	*frq*		
IPSILATERAL RATIOS	Dominant leg	H/Q_CONC_		***< 0.6***				***< 0.6***		
		60	0.57 ± 0.11	5			0.58 ± 0.05	5		
		180	0.70 ± 0.11				0.65 ± 0.10			
		240	0.66 ± 0.10				0.70 ± 0.12			
		H/Q_FUNC_		***< 0.7***				***< 0.7***		
		60	0.66 ± 0.15	**6**			0.66 ± 0.11	4		
	Non-dominant leg	H/Q_CONC_		***< 0.6***				***< 0.6***		
		60	0.57 ± 0.05	5			0.56 ± 0.06	5		
		180	0.64 ± 0.07				0.66 ± 0.10			
		240	0.64 ± 0.09				0.70 ± 0.13			
		H/Q_FUNC_		***< 0.7***				***< 0.7***		
		60	0.67 ± 0.10	5			0.62 ± 0.16	6		
		
BILATERAL FORCE DEFICIT				**≥10**	**≥15**	**≥20**		**≥10**	**≥15**	**≥20**
	Extension	conc								
		60	7.68 ± 6.98	1	2	0	3.89 ± 2.95	0	0	0
		180	3.17 ± 2.06	0	0	0	5.06 ± 2.84	0	0	0
		240	5.59 ± 5.14*	3	0	0	11.10 ± 5.60	2	2	0
		ecc								
		60	14.85 ± 10.53	0	1	3	10.72 ± 8.77	1	1	1
	Flexion	conc								
		60	10.04 ± 6.32	1	3	0	4.92 ± 2.74	1	0	0
		180	12.20 ± 10.79	2	1	2	6.18 ± 3.20	1	0	0
		240	10.04 ± 7.09	1	1	1	6.97 ± 4.83	2	0	0
		ecc								
		60	15.05 ± 13.46	2	0	3	8.95 ± 7.70	3	0	1

* p<0.05, different from WMJ;

H/Q_CONC_ – conventional hamstring to quadriceps ratio; H/Q_FUNC_ – functional hamstring to quadriceps ratio; conc – concentric action; ecc – eccentric action; 60, 180, 240°/s – angular velocity, DL – dominant leg, NL – non-dominant leg, SD – standard deviation, frq – frequency of risk values in individual evaluation (H/Q_CONC_ < 0.6; H/Q_FUNC_ < 0.7 at a velocity of 60°/s; BFD ≥10; ≥15; ≥20 expressed as percentage (%)).
